# SLEEP BARRIERS AND FACILITATORS IN AFRICAN AMERICAN FAMILY CAREGIVERS

**DOI:** 10.1093/geroni/igae098.2750

**Published:** 2024-12-31

**Authors:** Heba Aldossary, Glenna Brewster, Stephanie Griggs, Elliane Irani

**Affiliations:** Case Western Reserve University, Cleveland, Ohio, United States; Emory University, Atlanta, Georgia, United States; Case Western Reserve University, Cleveland, Ohio, United States; Case Western Reserve University, Cleveland, Ohio, United States

## Abstract

Cultural and historical experiences of African American family caregivers influence their overall health outcomes and can compound their risk for poor sleep health. Since there is inadequate representation of African American individuals in caregiving research, the existing literature is limited as it pertains to informing sleep intervention targets to address the unique needs of African American caregivers. This study aims to explore the barriers and facilitators to sleep health in African American family caregivers. To be eligible for this study, caregivers were required to provide care to an adult family member or friend with a chronic or disabling condition for a minimum of 6 months and self-identify as Black or African American. Twenty-four participants were recruited via community-based methods and participated in semi-structured in-person, telephone, or videoconference interviews. Transcribed interviews were analyzed using conventional content analysis. Participants were on average 58.0 (SD=10.1) years old, mostly female (83.3%) and caring for a parent (79.2%). Main sleep barriers included being alert to care recipient’s needs, general and caregiving-specific stress, existing health conditions such as anxiety or sleep apnea, and environmental factors such as room temperature and noise within and outside the home. Sleep facilitators included caregiving support, daytime activities for the care recipient and the caregiver, natural or pharmacological sleep aids, and relaxing activities at bedtime such as meditation, journaling, reading, and art-based activities. Half of our participants perceived the television before and/or during sleep as a facilitator. Our findings can inform the cultural adaptation of behavioral interventions to optimize sleep health.

